# Pressure Algometry Evaluation of Two Occlusal Splint Designs in Bruxism Management-Randomized, Controlled Clinical Trial

**DOI:** 10.3390/jcm10112342

**Published:** 2021-05-27

**Authors:** Bartosz Dalewski, Agata Kamińska, Paweł Kiczmer, Krzysztof Węgrzyn, Łukasz Pałka, Katarzyna Janda, Ewa Sobolewska

**Affiliations:** 1 Department of Dental Prosthetics, Pomeranian Medical University, 70-204 Szczecin, Poland; bar-tosz.dalewski@pum.edu.pl (B.D.); rpsobolewski@wp.pl (E.S.); 2 Outpatient Dental Clinic, Pomeranian Medical University, 70-204 Szczecin, Poland; agata.kaminsk@gmail.com (A.K.); kwegrzynstom@gmail.com (K.W.); 3 Department and Chair of Pathomorphology, Faculty of Medical Sciences in Zabrze, Medical University of Silesia, 40-055 Katowice, Poland; pawel.kiczmer@protonmail.com; 4 Private Dental Practice, 68-200 Żary, Poland; 5 Department of Human Nutrition and Metabolomics, Pomeranian Medical University, 24 Broniewskiego Street, 71-460 Szczecin, Poland; Katarzyna.Janda@pum.edu.pl

**Keywords:** bruxism, sleep disorder, algometer, pressure pain threshold, splint, grinding, clenching, canine guidance, TMD, occlusal appliance, masticatory muscle, masseter, temporalis

## Abstract

The aim of this pilot study was to evaluate the short-term effectiveness of two different occlusal devices and their impact on the pressure pain threshold (PPT) values among patients who reported to the Dental Prosthetics Outpatient Clinic of Pomeranian Medical University (Szczecin, Poland) and who were diagnosed with probable bruxism. Two groups were formed (A and B) to which patients were assigned randomly. Each group used a different occlusal splint for bruxism management. The occlusal appliance by Okeson, or the bimaxillary splint, was used overnight by each patient for 30 days of the study. The PPT was measured twice, at the first visit and after 30 days of using each occlusal device, with Wagner Paintest FPX 25 algometer. Bruxism was diagnosed based on data from the patient’s medical history and from the physical examination. Nocturnal Bruxism Criteria according to the International Classification of Sleep Disorders (Third Edition) was used for the patient’s evaluation. Results: similar pain factor (PF) reduction was observed in both the examined groups, regardless of the device used; canine guidance and no guidance were similarly effective in terms of increasing pain resilience.

## 1. Introduction

According to the glossary of Prosthodontic Terms, bruxism is described as the parafunctional grinding of teeth or an oral habit consisting of the repetitive, nonfunctional gnashing or clenching of teeth other than when chewing, which may lead to occlusal trauma [1]. Another definition of bruxism provided by the International Classification of Sleep Disorders Third Edition (ICDS-3) refers to it as a sleep-related movement disorder. Lobbezoo et al. [2] defined it as a repetitive jaw muscle activity characterized by the clenching or grinding of the teeth and/or by the bracing or thrusting of the mandible [3]. There are two forms of bruxism: sleep and awake [4]. By definition, awake bruxism occurs when the patient is aware of their jaw clenching. The most common symptoms of awake bruxism include an unpleasant noise made by tooth grinding, jaw muscle pain or stiffness, headache, TMJ noise, difficulty when moving the mandible, tooth hypersensitivity, tooth chipping and cervical defects [5]. Moreover, tongue indentation, bilateral masseter muscle hypertrophy with jaw muscle tenderness to palpation [5], TMJ palpation pain, as well as fracture or failure of dental restorations might be observed [6]. Its prevalence among the general population reaches between 20% and 31.4% [7,8]. On the other hand, sleep bruxism is reported by 8% of the general population and sound scientific evidence in terms of its’ etiology is still sparse [7,9]. Pain is one of the strongest negative effects a person can experience. As it is here located in the orofacial area, this condition makes it difficult, or even impossible, to perform everyday activities such as speech, eating or expressing emotions [10]. There were also several associations between clenching, orofacial pain and depression reported [11,12,13]. Hence, correlations between sleep bruxism, hypertension, sleep apnea and the cervical spine are the subjects of extensive investigations, yet without tangible evidence between these symptoms [14,15,16].

It has also been reported that a pressure algometer could be an effective tool in the screening and evaluation of patients with muscle pain, since tissue sensitivity can be measured [17,18,19,20,21,22]. As shown above, there have been many attempts to evaluate bruxism management using a plethora of devices, with a mixed ratio of success [23,24,25,26].

To date, conclusions regarding pressure algometry are inconsistent. The lack of accuracy and repeatability of the research designs makes it impossible to apply them in everyday practice. Considering the above-mentioned data, we decided to attempt to design a repeatable testing scheme with a pressure algometer and to find out what the changes in the PPT of the orofacial muscles are after using two types of occlusal splints. A pressure algometer was used for a reliable presentation of the results. We hypothesized that PPT measurements might be affected by a splint design. The aim of this pilot study was to compare how different splint designs may alter the pain threshold in chosen muscles and TMJ pressure points in patients with probable bruxism.

## 2. Materials and Methods

**Figure 1:**
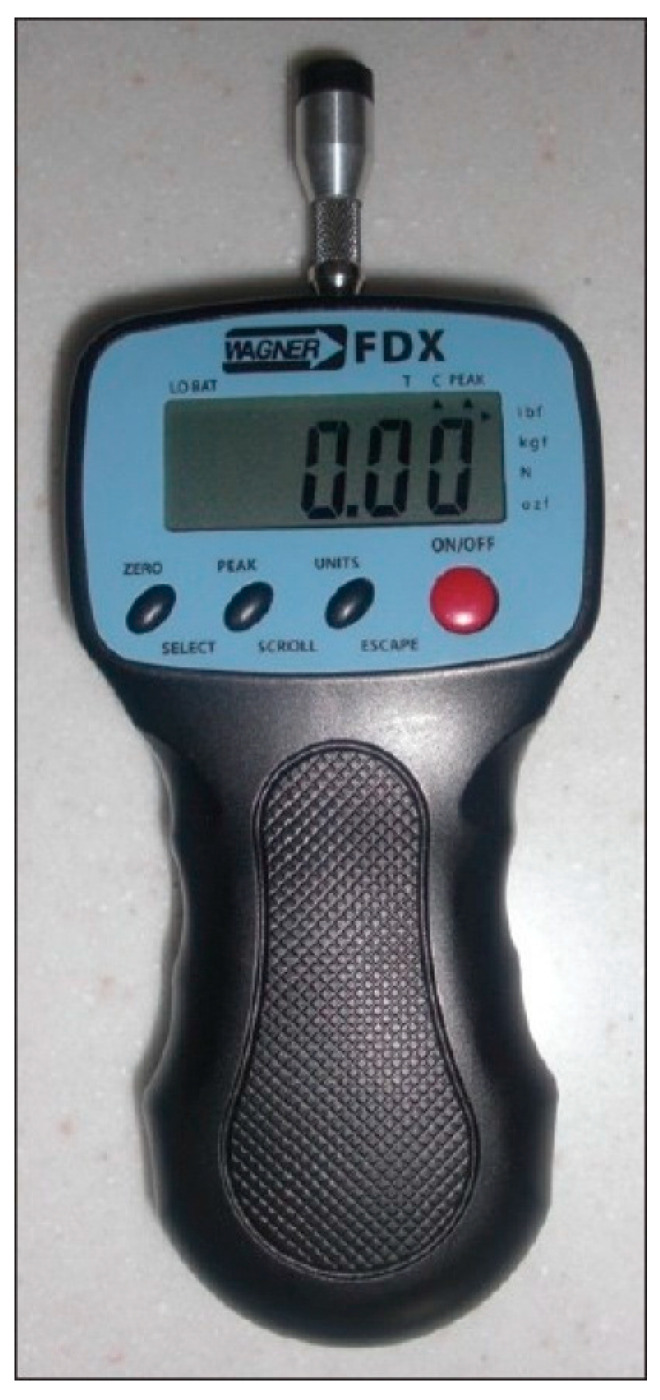
Wagner Paintest FPX 25.

The trial was registered at ClinicalTrials.gov (identifier NCT04733573). The study was conducted among patients who reported to the Dental Prosthetics Outpatient Clinic of Pomeranian Medical University, Szczecin, Poland and who had probable bruxism diagnosed. The study was conducted according to the guidelines of the Declaration of Helsinki and approved by the Institutional Review Board (or Ethics Committee) of Pomeranian Medical University in Szczecin (protocol code KB-0012/49/10). We included 30 patients of both sexes, mean age 24.8 years. Two study groups were formed (A and B), with 15 patients each. Patients were assigned randomly to one of the groups. Each group used a different occlusal appliance (occlusal appliance by Okeson for group A or bimaxillary splint for group B) for 30 days during sleep. Sealed, opaque envelopes were used for randomization. PPTs were measured at the first day of study and after 30 days of using each occlusal device, with a Wagner Pain test FPX 25 algometer (Figure 1). When patients needed analgesics, ibuprofen was administered orally during the study, not to exceed 800 mg per day.

### 2.1. Inclusion Criteria

Patients aged 18–65, with all occlusal support zones preserved (natural dentition or fixed partial dentures), and with probable bruxism found according to the formal criteria for sleep-related bruxism according to the International Classification of Sleep Disorders, Third Edition (ICSD-3), which lists [1] the presence of regular or frequent tooth-grinding sounds occurring during sleep, and the presence of one or more of the following clinical signs: abnormal tooth wear consistent with the above reports of tooth grinding during sleep, transient morning jaw muscle pain or fatigue, and/or temporal headache, and/or jaw locking upon awakening consistent with the above reports of tooth grinding during sleep.

### 2.2. Exclusion Criteria

**Figure 2:**
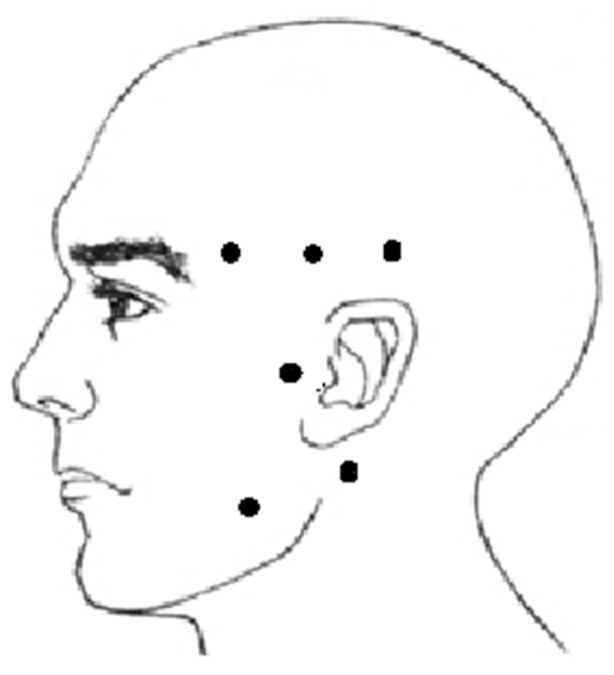
PPT measurement points.

Unrestored tooth losses; patients wearing removable dentures of any kind; psychiatric conditions undermining/precluding occlusal splint usage; prior occlusal splint therapy; taking oral contraceptives or hormone therapy; taking antidepressants; metabolic or rheumatic diseases; lack of mandible orthopedic stability; use of anti-inflammatory or analgesic drugs within 3 months before the examination; pregnancy; active or chronic inflammation in the area of face, head or neck; patients undergoing an orthodontic treatment; patients who have ever used botulinum toxin type A injections within masticatory muscle area [27]. PPT measurement points are shown in Figure 2.

Points within the following muscles were examined bilaterally [28,29]:

- masseter muscle (M)—point on maximal muscle convexity during maximal intercuspation;
- anterior belly of temporal muscle (MTA)—point on maximal muscle convexity during maximal intercuspation;
- medial belly of temporal muscle (MTP)—3 cm distally from the anterior belly examination point in horizontal plane;
- posterior belly of temporal muscle (MTM)—6 cm distally from the anterior belly examination point in horizontal plane;
- point in the projection of the temporomandibular joint (TMJ)—point at the height of the mandibular condyle congruent with hinge axis;
- sternocleidomastoid muscle (SCM)—point 1 cm downwards from the muscle insertion on the mastoid process along the course of the muscle.

### 2.3. PPT Measurement Technique and Principles

Initially, each patient was introduced to the study and familiarized with the PPT testing by examination on the outer surface of the first metacarpal bone (MB) and at the C7 vertebra level (control points). Patients were examined in an upright, stabilized sitting position. Patients had to inform the examiner when the feeling of pressure first changed into a feeling of pain. The pain threshold level is the minimum pressure that causes pain. The test started at 0 (kg/s) and the pressure was increased at a rate of 1 kg/s. All examinations were made by the same person from the research team. PPT test points were examined twice at each visit and the measurements were recorded. During the study duration no drop-outs or adverse effects of the splints were recorded.

To manufacture both types of splints, maxillary and mandibular impressions were made using alginate (impression material Kromopan, Lascod, Italy). Centric relation was registered with a sliding guide (Amann Girrbach, Bregenz, Austria) in each case. Casts were made of grade 3 plaster (A-III Yellow Dental Gypsum, VDental, Bielawa, Poland), and each splint was fabricated using a facebow and an articulator (Artex CR, Amann Girbach, Bregenz, Austria) and was manufactured from a self-polymerizing acrylic resin (Villacryl, BadiaPolesine, Italy).

**Figure 3:**
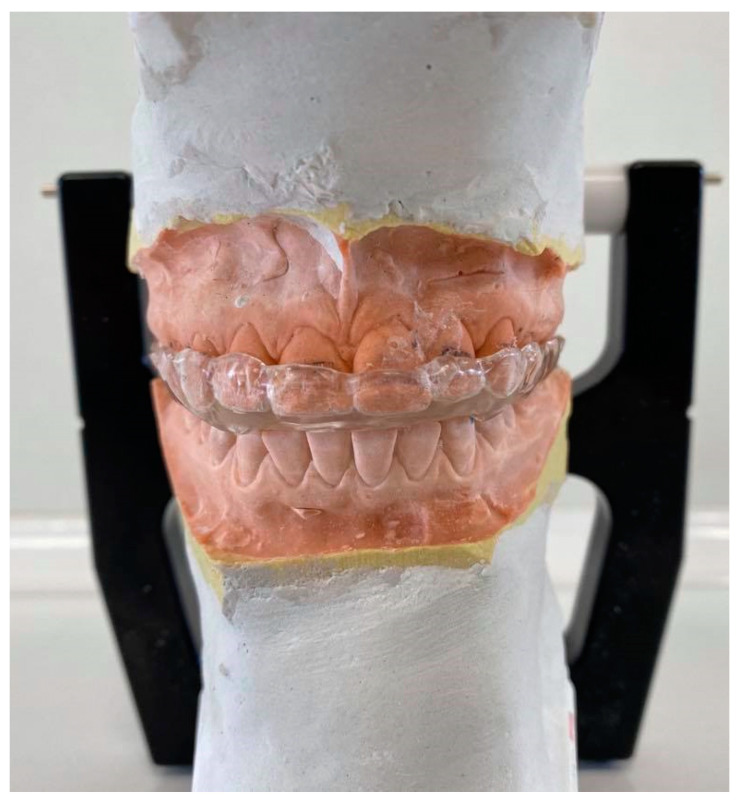
An occlusal appliance.

An Okeson’s stabilization (group A) splint is mainly made on the upper arch [10]. While using it, the mandible heads are positioned in a stable musculoskeletal position. Lower teeth contact the splint evenly and simultaneously. Canine guidance ensures the dislocation of the posterior teeth during eccentric movements. On the vestibular surfaces of teeth, the splint reaches up to 1/3 of the teeth height (Figure 3) [10].

**Figure 4:**
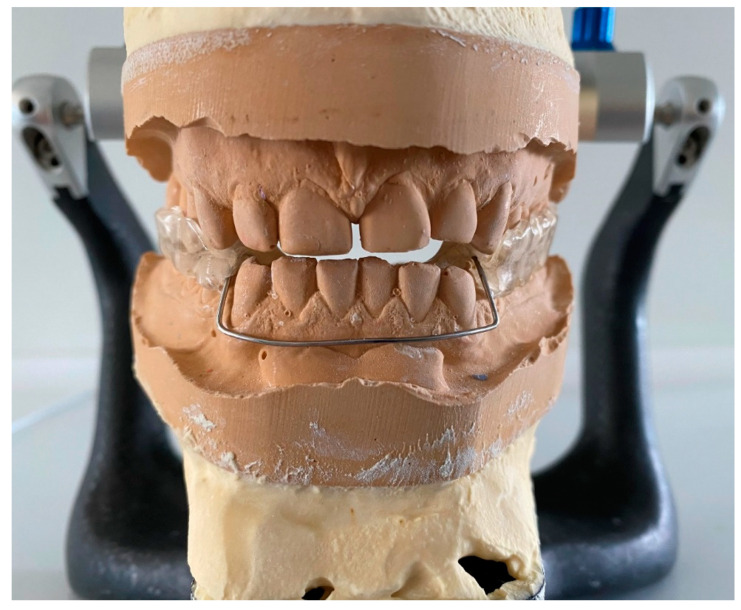
A bimaxillary splint.

In group B, the bimaxillary splints, with the lower arch connecting both arches, were prepared. While using it the mandible heads are positioned in a stable musculoskeletal position. It does not provide any guidance, but only increases the distance between the bone bases of the maxilla and mandible. The wire arch stretches in the vestibule between the lower canines (Figure 4).

At the time of splint delivery, the patients received written instructions on how to maintain each splint type.

### 2.4. Applied Bruxism Diagnostic Criteria

For the purpose of this study, probable bruxism was diagnosed on the basis of data from the medical interview (Appendix A Questionnaire 1) and from the physical examination [3]. Nocturnal Bruxism Criteria according to the International Classification of Sleep Disorders (Third Edition) was used for the patients evaluation (Appendix A Questionnaire 2) [2,30].

### 2.5. Statistical Analysis

Data from the evaluation were presented as the mean ± standard deviation (SD) (Table 1). Assessment of correlations between the variables was performed using Pearson’s coefficient and presented as a heatmap with hierarchical clustering obtained using Seaborn library for Python [31,32]. To evaluate the differences between group A and group B before and after treatment, a non-parametric ANOVA for repeatable measurements was used. To perform the analysis, we used the nparLD package [33]. Principal components analysis was performed to reduce the amount of data obtained during the study. Analysis was performed using RStudio software (Integrated Development for R. RStudio, PBC, Boston, MA, USA). Principal component analysis with varimax rotation was performed to reduce the amount of variables and clarify the influence of both splints on pain sensations. The obtained factor values were used as a new variable named “pain factor” in further analyses. *p* values ≤ 0.05 were considered significant.

## 3. Results

**Figure 5:**
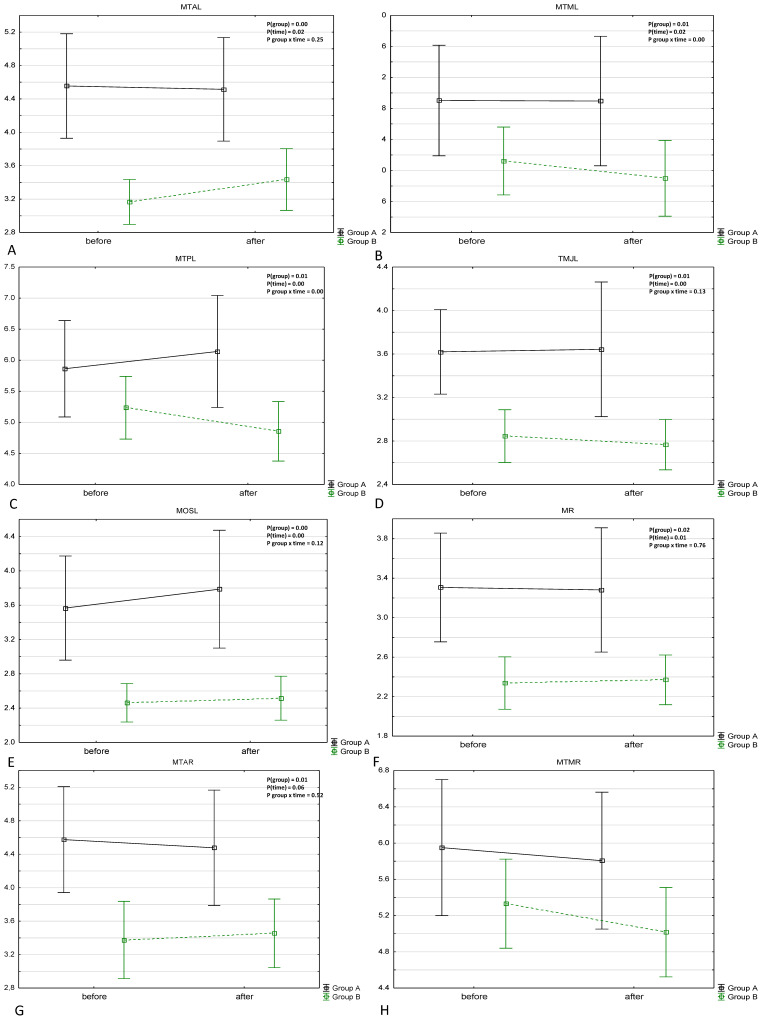

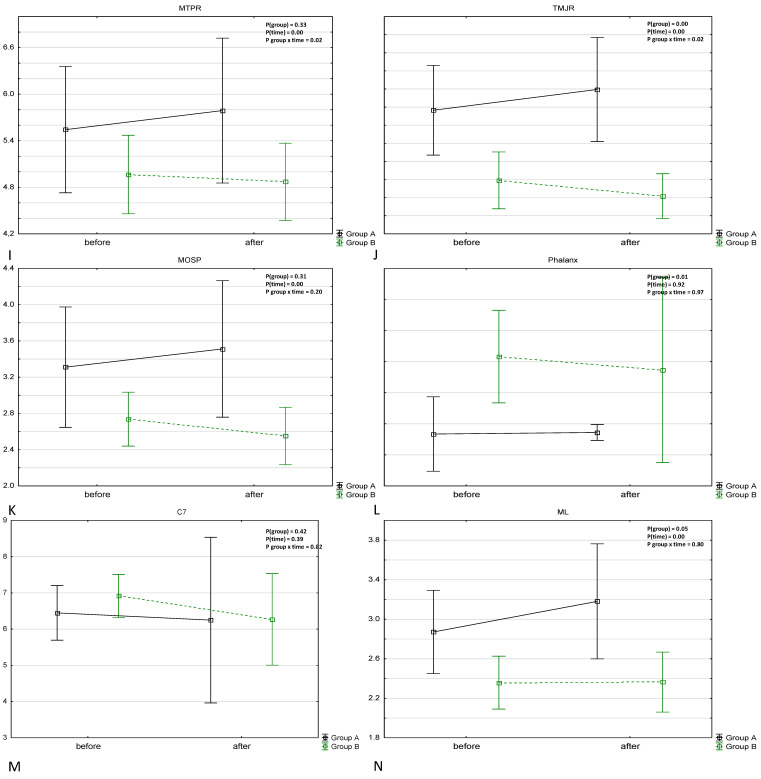
Results of algometric measurements among the subjects presented as box and whiskers plot. A: MTAL algometric measurements, B: MTML algometric measurements, C: MTPL algometric measurements, D: TMJL algometric measurements, E: MOSL algometric measurements, F: MR algometric measurements, G: MTAR algometric measurements, H: MTMR algometric measurements, I: MTPR algometric measurements, A: TMJR algometric measurements, K: MOSP algometric measurements, L: Phalanx algometric measurements, M: C7 algometric measurements, N: ML algometric measurements. All data are presented as Mean +/− SD. Results of non parametric analysis for repeatable measurements (nparLD) are presented in the right corner of each plot.

A significant group effect was found in almost all measurements excluding MTMR, MTPR, SCM and C7. Group A had initially higher readings in all measurements than group B. A significant time effect was found in most algometer readings, excluding MTAR (examined) and MB and C7 (control points), and in all the other measurements a significant increase in PPT values was noted. A significant time and group effect interaction was found in MTML and MTPL readings, while in group B the increase in these parameters was greater than in group A. The results are presented in Table 2 and Figure 5.

### Figures and Tables

**Figure 6:**
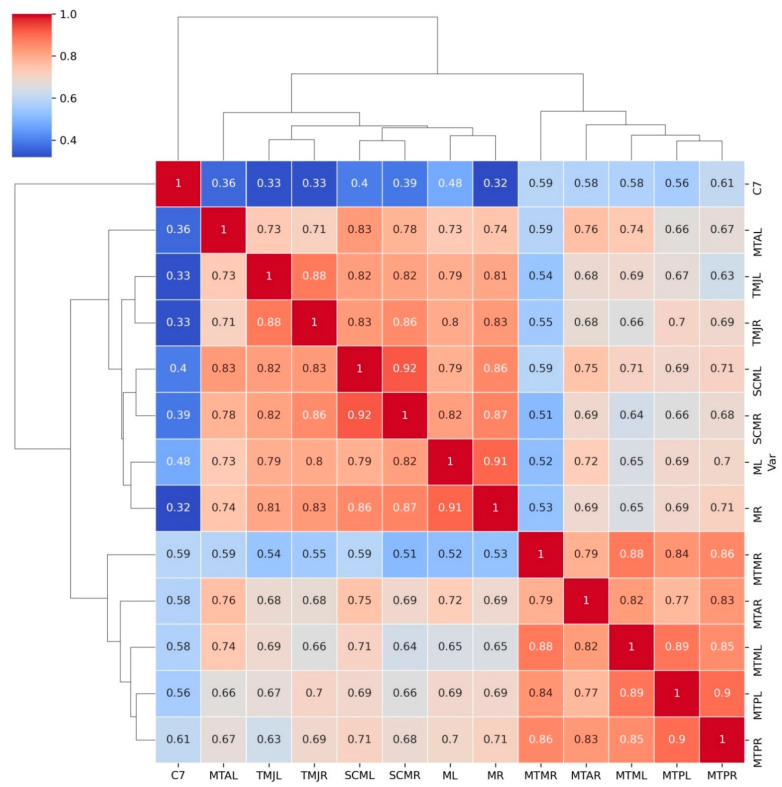
Graphical interpretation of correlations between the algometric measurements grouped using hierarchical clustering (Pearson’s correlation coefficients presented in squares). More detailed correlations may be found in Figure 5.

Significant correlations between algometric measurements were found and are presented as a hierarchical clustered heatmap below (Figure 6).

Due to the strong positive correlations between most of the measurements, principal component analysis was performed. Most elements showed significantly positive correlations, which are presented in Figure 5. The results indicated that one principal component corresponded to 76% of the overall variance. This obtained factor was described as “pain factor” and its values are presented in Table 2.

**Figure 7:**
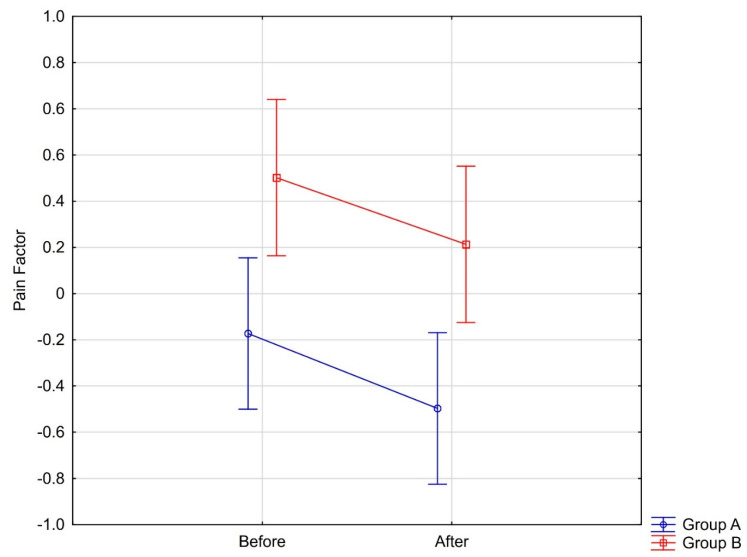
Pain factor changes among groups during experiment.

The values of the “pain factor” (PF) for each case were further analyzed. Significant time and group effects were found for PF. Both groups presented a drop in pain sensations after treatment; however, this change was similar in both examined groups. Group A had initially lower pain sensations (Figure 7).

## 4. Discussion

Our study showed a similar PF reduction in both examined groups, regardless of the device used. Occlusal appliance therapy is one of several bruxism management methods [34]. Myofascial pain, as a subjective sensation, is often evaluated with a Visual Analogue Scale (VAS). Herein, our findings were consistent with the study by Keskinruzgar et al., who incorporated thirty-four patients. Clinical examination was performed in the search for the presence of shiny dental restorations, dental abrasion, hypertrophy in masseter muscles, and also palpation-induced pain in masseters. Patients were divided into two groups: the first one received kinesio taping, while the second received an occlusal splint for the maxilla. Musculoskeletal pain was measured according to the VAS scale; mouth opening measurements and PPT for temporal and masseter muscle were bilaterally recorded before treatment, and at the first and fifth weeks, consecutively. They concluded that the VAS values were significantly lower, while PPT was significantly higher, at the fifth week in both examined groups compared to the initial assessment of both muscles [35]. Beril et al. [36] compared the effectiveness of occlusal splints and transcutaneous electrical nerve stimulation (TENS). They selected patients who were aware of grinding, with clinically detected abrasion, muscle tenderness to palpation and myofascial trigger points (MTRPs) in temporal and/or masseter muscle. After randomization, 30 patients were divided into two examined groups: one with the mandibular occlusal splint, while the second received TENS. PPT values were measured on trigger points in both muscles before and after treatment, with a follow-up after 1 month. All these PPT measurements were higher after treatment and after 1 month; however, there was no statistical significance between the groups [36]. This is also partly consistent with our findings, as similar results were obtained. Other attempts of evaluating intraoral devices have been undertaken, as the effect of the mandibular advancement device (MAD) was evaluated by Alessandri-Bonetti et al. For this study 27 patients with obstructive sleep apnea (OSA) were selected and compared to 27 healthy patients, with a matching age and sex, as controls. PPTs were measured before treatment, after 15 days and after 6 months. The initial PPT readings were similar for both groups, while after 15 days they decreased significantly for masseter and temporalis in the MAD group. Nevertheless, follow-up examination after six months did not confirm any difference in PPT, which sparked a concluding idea suggesting physiological adaptation and regression to the mean. Nilner et al. [37] compared a stabilization appliance with a prefabricated relax appliance in patients with TMD based on research diagnostic criteria (DC/TMD) [38]. Out of 65 patients, two randomly distributed groups were formed, each with a different splint. Significant changes in PPT were found for masseter on the right side, at the 6th week, and for both temporal muscles at the 10th week when compared to measurements before treatment [37]. A similar study to ours, in terms of outcome, was conducted by Öz et al. They evaluated myofascial pain in a group of 40 patients, in which DC/TMD criteria were used for inclusion. The studied group received low-level laser therapy and was compared to the use of an occlusal splint (control group). Patients in the control group were asked to use the splint for 3 months, 24 h/d; after this time, the outcomes were measured and compared with those taken before the treatment. The results showed a significant decrease in tenderness to muscle palpation for both groups with similar results for PPT [38]. Our results showed that both occlusal splint designs increased PPT during the second evaluation (excluding control points), and a similar effect on the change of PPT in both groups was achieved. Moreover, the effects of the occlusal splint therapy, measured in reduction of myofascial pain, were reported by Gomes et al. [39] and Noguchi et al. [40]. These may partially explain our results. Studies by Fatella et al. [41] and Silva et al. [42] both reported lower PPT values for patients with myofascial pain than in the healthy controls; our study also noted initially lower PPT readings in comparison with after 30 days of treatment. The comparable results of both appliances were not in compliance with our hypothesis. One possible explanation relates to the change in parafunctional behavior.

Differences in PPT measurements between the studies may be related to inconsistencies in PPT measurement points selection. The measurement points in this study were selected in accordance with meta-analysis performed by Kaminska et al. [29].

As demonstrated in the study of Reichardt et al. [43], a change in occlusal guidance relates to different grinding patterns. This probably leads to different muscle activity and allows the affected muscles’ regeneration [10]. Our results are partly consistent with findings achieved by Rugh et al. [44], who proved that splints with canine and molar guidance did not give rise to different results in pain or clinical examination.

## 5. Limitations of the Study

The present study solely used PPT values for the evaluation of both appliances in bruxism management [29]. The acquired results might also be improved by increasing the sample size, yet the follow-up period might be longer. The placebo effect, or sham splint use, might also add more power to future PPT studies. Different algometer models might also be implemented in the future, possibly adding more examined points within the head and neck area. More studies with unified bruxism diagnostics criteria, well-defined PPT measurement points, specific occlusal appliances and longer follow-up are needed.

## 6. Conclusions

This randomized study showed a similar PF reduction in both examined groups, regardless of the device used; canine guidance and no guidance were similarly effective in terms of increasing pain resilience at the examined points. During the treatment period, the PT changes did not statistically differ among the two groups, thus it may be concluded that both splint designs can be effective in altering PPT in patients with diagnosed bruxism.

## Figures and Tables

**Table 1:** Results of algometric measurements among the subjects. Significant *p* values are highlighted in bold.

Variable	Group A				Group B				*p*		
	Before		After		Before		After		Group	Time	Group × Time
	Mean	SD	Mean	SD	Mean	SD	Mean	SD			
ML	2.85	1.19	3.20	1.60	2.29	0.70	2.43	0.82	**0.05**	**0.00**	0.80
MTAL	4.12	1.36	4.95	1.94	3.22	0.76	3.38	0.98	**0.00**	**0.02**	0.25
MTML	5.59	1.93	6.20	2.31	4.63	0.88	5.39	1.43	**0.01**	**0.00**	**0.01**
MTPL	5.84	2.16	6.17	2.49	4.55	1.00	5.54	1.43	**0.01**	**0.00**	**0.00**
TMJL	3.40	1.11	3.87	1.66	2.67	0.68	2.94	0.56	**0.00**	**0.00**	0.13
MOSL	3.54	1.61	3.82	1.96	2.33	0.56	2.65	0.69	**0.00**	**0.00**	0.12
MR	3.12	1.41	3.47	1.83	2.27	0.65	2.44	0.72	**0.02**	**0.01**	0.76
MTAR	4.32	1.68	4.74	1.96	3.40	1.15	3.43	1.19	**0.01**	0.06	0.52
MTMR	5.76	2.07	6.00	2.10	4.91	1.04	5.44	1.52	0.22	**0.00**	0.15
MTPR	5.34	2.10	6.00	2.69	4.59	1.04	5.25	1.52	0.33	**0.00**	**0.02**
TMJR	3.53	1.60	3.83	1.36	2.41	0.77	3.00	0.62	**0.00**	**0.00**	**0.02**
MOSP	3.23	1.92	3.60	2.00	2.44	0.84	2.85	0.77	0.31	**0.00**	0.20
phalanx	5.31	1.79	5.37	1.32	6.61	1.59	6.46	1.82	**0.01**	0.92	0.97
c7	6.30	1.97	6.58	1.99	6.55	1.14	7.03	1.58	0.42	0.39	0.82
pain factor	−0.17	1.08	−0.50	1.32	0.50	0.45	0.21	0.56	**0.00**	**0.00**	0.10

**Table 2:** Loadings of variables used to component interpretation.

Variable	Factor 1 (Pain Factor)
ML	−0.87
MTAL	−0.86
MTML	−0.87
MTPL	−0.87
TMJL	−0.87
MOSL	−0.91
MR	−0.89
MTAR	−0.88
MTMR	−0.78
MTPR	−0.88
TMJR	−0.88
MOSP	−0.89

## Data Availability

The data presented in this study are available on request from the corresponding author. The data are not publicly available due to sensitive information.

## Abbreviations

ML	left masseter muscle
MTAL	left temporal muscle, anterior belly
MTML	left temporal muscle, medial belly
MTPL	left temporal muscle, posterior belly
TMJL	left temporomandibular joint
SCML	left sternocleidomastoid muscle
MR	right masseter muscle
MTAR	right temporal muscle, anterior belly
MTMR	right temporal muscle, medial belly
MTPR	right temporal muscle, posterior belly
TMJR	right temporomandibular joint
SCMR	right sternocleidomastoid muscle
MB	the outer surface of the first metacarpal bone
C7—C7	vertebra level

## Appendix A

Questionnaire 1. Questionnaire for detecting Bruxers.

1. Has anyone heard you grinding your teeth at night?
2. Is your jaw ever fatigued or sore in the morning?
3. Are your teeth or gums ever sore on awakening in the morning?
4. Do you ever experience temporal headaches on awakening in the morning?
5. Are you aware of grinding your teeth during the day?
6. Are you ever aware of clenching your teeth during the day?

Questionnaire 2. Nocturnal Bruxism Criteria according to International Classification of Sleep Disorders, Third Edition

- The patient reports or is aware of tooth-grinding sounds or tooth clenching during sleep.
- One or more of the following is present:
  - Abnormal wear of teeth;
  - Jaw muscle discomfort, fatigue, or pain and jaw lock upon awakening;
  - Masseter muscle hypertrophy upon voluntary forceful clenching.
- The jaw muscle activity is not better explained by another current sleep disorder, medical or neurological disorder, medication use, or substance use disorder.
